# Protein and Essential Amino Acids to Protect Musculoskeletal Health during Spaceflight: Evidence of a Paradox?

**DOI:** 10.3390/life4030295

**Published:** 2014-07-11

**Authors:** Kyle J. Hackney, Kirk L. English

**Affiliations:** 1 Department of Health, Nutrition, and Exercise Sciences, North Dakota State University, Fargo, ND 58102, USA; 2 Exercise Physiology and Countermeasures Laboratory, JES Tech, Houston, TX 77058, USA; E-Mail: kirk.english-1@nasa.gov

**Keywords:** spaceflight, exercise countermeasures, skeletal muscle, bone, amino acids, leucine, disuse, unloading

## Abstract

Long-duration spaceflight results in muscle atrophy and a loss of bone mineral density. In skeletal muscle tissue, acute exercise and protein (e.g., essential amino acids) stimulate anabolic pathways (e.g., muscle protein synthesis) both independently and synergistically to maintain neutral or positive net muscle protein balance. Protein intake in space is recommended to be 12%–15% of total energy intake (≤1.4 g∙kg^−1^∙day^−1^) and spaceflight is associated with reduced energy intake (~20%), which enhances muscle catabolism. Increasing protein intake to 1.5–2.0 g∙kg^−1^∙day^−1^ may be beneficial for skeletal muscle tissue and could be accomplished with essential amino acid supplementation. However, increased consumption of sulfur-containing amino acids is associated with increased bone resorption, which creates a dilemma for musculoskeletal countermeasures, whereby optimizing skeletal muscle parameters via essential amino acid supplementation may worsen bone outcomes. To protect both muscle and bone health, future unloading studies should evaluate increased protein intake via non-sulfur containing essential amino acids or leucine in combination with exercise countermeasures and the concomitant influence of reduced energy intake.

## 1. Introduction

Humans possess an intrinsic desire to explore the unknown. The initial conquest of space occurred on 12 April 1961 with Soviet Union cosmonaut Yuri Gagarin’s solo orbital flight. Less than a month later on 5 May 1961, Alan Shepard became the first American in space. The remainder of the decade was filled with humans traveling to space, climaxing on 20 July 1969 with the Apollo 11 Moon landing. However, all of the 1960s space missions were less than two weeks in duration. In 1971, the Soviet Union launched Salyut 1, its first orbiting space station which was later manned for 23 days. Over the next decades, the duration of missions grew increasingly longer as America and the Soviet Union each launched orbiting space stations; the American Skylab 4 mission lasted 84 days in 1973–1974 and the Soviet Salyut 6 hosted a 185 day mission in 1980. This was followed by the Russian space station Mir which was manned for the majority of its time in orbit from 1986–2000. From 1981–2011, the American Space Shuttle made regular flights lasting 2–17 days and in collaboration with international partners, transported crewmembers to the International Space Station (ISS) for stays of ~6 months after the turn of the millennium. 

As the duration of microgravity exposure is increased and manned missions beyond low earth orbit (e.g., to Mars) are once again considered, the potential impact on musculoskeletal physiology is substantial. Specifically, losses in muscle strength, power, and endurance are problematic both from an operational and individual standpoint. Operationally, high levels of muscle function are needed both during spaceflight tasks (e.g., during extravehicular activity to free jammed hardware, or to move large mass/high inertia objects) and in the event of an emergency egress from a vehicle. An emergency egress in a gravitational environment would likely involve high force/power activities like opening a hatch, raising oneself and other crewmembers out of the vehicle, and running from the vehicle, all of which may be performed while wearing a space suit with a mass of >100 kg [1]. From an individual health perspective, upon return to Earth’s gravity, astronauts may be at increased risk for falls, injury from an accident, osteoporosis [2], and/or experience difficulty with activities of daily living [3]. 

This paper addresses the importance of preserving musculoskeletal health during long-duration spaceflight and examines the seemingly paradoxical effects of amino acids on muscle and bone. Recommendations for future nutrition and exercise countermeasures to prevent spaceflight deconditioning will also be detailed.

## 2. Musculoskeletal Health and Spaceflight

### 2.1. Skeletal Muscle

In the absence of gravitational loading, skeletal muscle enters a *de facto* state of disuse [4,5]. Given the complexity of spaceflight research and the few crewmembers available to study, data on the effects of spaceflight on human muscle are relatively sparse. Regardless, the loss of skeletal muscle mass has been a consistent, yet unwelcomed, feature of spaceflight. A synthesis of the available data (Table 1) suggests that the rate of muscle mass loss is not linear across the duration of a mission and that the greatest losses occur in the antigravity muscles (lower back, abdominals, thighs, and lower legs) [4]. Significant atrophy of the knee extensors (−5% to −15%), knee flexors (−5% to −14%), plantar flexors (−6% to −15%) and muscles of the intrinsic lower back (−10%) has been documented even during short-duration missions of 9–17 days [6,7,8]. During longer duration missions on Mir (115–197 days) and the ISS (161–192 days), it appears that the muscles of the intrinsic lower back (rotatores, multifidus, semispinalis, spinalis, longissimus, iliocostalis, −16%) and the ankle plantar flexors (soleus, gastrocnemius, −13% to −17%) are the most influenced by microgravity exposure (or the countermeasures for these muscles groups are not as efficacious) [8,9]. Of the plantar flexors, the soleus shows greater losses in fiber size and force production than the gastrocnemius [9,10].

A decrease in the rate of muscle protein synthesis (−46%) appears to be the primary mechanism underlying unloading-induced muscle atrophy [11]. This causes rapid decreases in muscle fiber cross-sectional area (CSA) with short-duration spaceflight eliciting greater losses in type II fibers while type I fibers experience greater atrophy after long-duration missions [4]. For example, Edgerton *et al.* [12] showed that the CSA of vastus lateralis muscle fibers were 16%–36% smaller after 11 days of spaceflight, with the reductions in type IIb > type IIa > type 1. Similar observations were made in soleus (−26% in type IIa, −15% in type 1) [13] and lateral gastrocnemius muscle fibers (−10% in type IIa, −7% in type I) [14] after 17 days of spaceflight. However, more recent analyses from long-duration ISS crewmembers’ plantar flexors suggest that fiber mass was most affected in the following order: soleus type I (−33%) > soleus type II (−29%), gastrocnemius type I (not reported), and gastrocnemius type II (−5%) [10]. The authors noted that there is a direct association between pre-flight fiber size and the degree of atrophy (r = 0.87), suggesting that larger fibers lose the most size during microgravity exposure [9,10]. The functional consequences of these negative adaptations are reduced force, contractile velocity, and power [10], which ultimately result in less energy transfer to the tendon and skeleton for joint movement.

### 2.2. Bone

Another significant maladaptation to long-duration spaceflight is a loss of bone mineral from the skeleton (Table 2) [15,16,17,18,19,20,21,22]. Data from the three Skylab flights suggest that urinary calcium levels increased approximately 80% (169–306 mg∙d^−1^ on average) compared to pre-flight during the first month of microgravity exposure and remained elevated for the duration of the mission [23]. Net intestinal absorption of calcium also declined approximately 7%, while plasma total calcium showed a small increase [23]. Markers of bone resorption (urinary hydroxyproline) were also elevated by 34%. Ultimately, bone mineral loss was reported in weight-bearing bone such as the calcaneus, but not in non-weight-bearing bones (e.g., the radius or ulna).

**Table 1:** Human Spaceflight and Skeletal Muscle Adaptations.

Author	Yr	Space Era	N (Gender)	Flight Duration	Exercise Countermeasures	Highlighted Adaptations
Thorton [24]	1974	Skylab2	3(M)	28 d	Bicycle Erg	M.Volume (Girth Circ.): ↓4.5% Legs; Strength. (Isok): ↓20% Legs
Thorton [24]	1974	Skylab 3	3(M)	59 d	Bicycle Erg., MK-I, MK-II	M.Volume (Girth Circ.): ↓5% Legs, Strength (Isok): ↓20% Legs
Thorton [24]	1974	Skylab 4	3(M)	84 d	Bicycle Erg., MK-I, MK-II, Thorton Passive Treadmill	M.Volume (Girth Circ): ↓2% Legs, Strength. (Isok): ↓7% Legs
Leblanc [7]	1995	STS	4(2M2W)	8 d	Shuttle Erg	M.Volume (MRI):↓6.3%, Gastr, ↓3.9% Ant. Calf. ↓8.3% Ham, ↓6.0% Quad, ↓10.3% L.Back
Edgerton [12]	1995	STS	8(5M3W)	5–11 d	Shuttle Treadmill, LBNP	F. CSA: ↓11% type I &, ↓24% type II VL in 5 d, ↓16%–36% type II loss > type I in 11 d
Widrick [13]	1999	STS/Mir	4(M)	17 d	NR	F. Diam/: ↓8% in Sol. type I
Akima [6]	2000	STS	3(?)	9–16 d	NR	Volume (MRI): ↓5.6% to 15.4% KE, ↓8.6% to 14.1% KF, ↓8.4% to 15.9% PF
Leblanc [8]	2000	STS/Mir	4(M)	17 d	NR	Volume (MRI): ↓10% AE & IB, ↓5%–7% Quad & Psoas, ↓3% in Ham & AL
Leblanc [8]	2000	STS/Mir	16(15M1W)	115–197 d	NR	Volume (MRI): 14.6% AL, ↓16.9% Gast, ↓16.8% Sol, ↓10.1% Quad, ↓12.7% Ham, ↓15.9% IB, and ↓4.4% Psoas
Riley [25]	2000	STS	4(M)	17 d	NR	F: A Band ↓17% Filaments, ↑9% Short Filaments
Koryak [26]	2001	Mir	7(M)	6 mo	NR	Strength (Isom): ↓42% TS, Strength: (Evoked Forces): ↓26% P_o_ TS, ↑15% P_t_ TS
Trappe [14]	2001	STS	4(M)	17 d	NR	F.CSA: ↓7.4% Type I Gast, ↓10.1 Type IIa Gast, ↓7.4 Type I Sol. ↔ Calf MVC or Force Velocity Relationship
Lambertz [27]	2001	Mir	14(M)	90–180 d	Cycle Erg., Treadmill	Strength (Isom): ↓2%–30% PF in 12 of 14 subjects
Narici [28]	2003	STS	4(M)	17 d	NR	M.CSA (Girth Circ.): ↓8% Calf., Strength (Evoked Forces) ↓24% Peak Twitch; ↓22% Tetanic Force at 50 Hz; ↓19.5% Specific Forces at 50 Hz of TS
Tesch [29]	2005	STS	4(M)	17 d	NR	M. CSA (MRI): ↓8% KE & Gluteal. Strength (Isom): ↓10% KE, Strength (Isok): ↓9% KE (CON), 11% KE (ECC)
Trappe [9]	2009	ISS	9(?)	6 mo	CEVIS, TVIS, VELO, iRED	M.Volume (MRI): ↓13% Calf, ↓15% Sol, ↓10% Gast. Calf Peak Power ↓32%, Force-Velocity ↓20%–29% across 30–300°/sec
Fitts [10]	2010	ISS	9(?)	6 mo	CEVIS, TVIS, VELO, iRED	F.CSA: ↓33% type 1 Sol > type II Sol > type I Gast. > type II Gas
Gopalakrishnan [30]	2010	ISS	4(M)	181 d	CEVIS, TVIS, VELO, iRED	M.Volume (MRI): ↓10 calf, ↓4 thigh
Smith [31]	2012	ISS	8(6M2W)	160 d	CEVIS, TVIS, iRED	Lean mass (DEXA): ↓2% total body
Smith [31]	2012	ISS	5(3M2W)	134 d	CVIS, TVIS, ARED	Lean mass (DEXA): ↑3% total body

STS = Shuttle transport system, ISS = International space station, CEVIS = Cycle ergometer with isolation system, TVIS = Treadmill with isolation system, iRED = Interim resistive device, VELO = Russian velociped bicycle exercise device, MRI = Magnetic resonance imaging, CSA = Cross-sectional area, Pec/Lats = Pectoralis major/lattisimus dorsi, Gastr = Gastrocnemius, Ant = Anterior, Ham = Hamstring, Quad = Quadriceps, VL = Vastus lateralis, Sol. = Soleus, KF = Knee flexors, KE = Knee extensors, PF = Plantar flexors, IB = Intrinsic back, TS = Tricpes surea, Circ. = Circumference, Isok = Isokinetic, Isom = Isometric, Erg = Egometer, LBNP = Lower body negative pressure, F. = Fiber, M. = Muscle, Diam. = Diameter, CON = Concentric, ECC = Eccentric, MVC = Maximal voluntary contraction, d = Days, Yr. = Year, mo = months, M = Men, W = Women. NR = Not reported.

**Table 2:** Human Spaceflight and Skeletal Adaptations.

Author	Yr	Space Era	N (Gender)	Flight Duration	Exercise Countermeasures	Highlighted Adaptations
Whedon [23,32]	1976	Skylab 2	3M	28 d	Bicycle Erg	↑Urinary calcium, ↑hydroproxyproline, ↑nitrogen and phosphorus excretion
Whedon [23,32]	1976	Skylab 3	3M	59 d	Bicycle Erg., MK-I, MK-II	↑Urinary calcium, ↑hydroproxyproline, ↑nitrogen and phosphorus excretion
Whedon [23,32]	1976	Skylab 4	3M	84 d	Bicycle Erg., MK-I, MK-II, Thorton Passive Treadmill	↑Urinary calcium, ↑hydroproxyproline,↑nitrogen excretion, phosphorus excretion
Stupakov [33]	1984	Salyut 6	8(NR)	75–184 d	KTF and penguin suit	Calcaneus BMD (Photon absorptiometry): ↓0.9% to19.8%
Organov [34]	1991	Salyut 7	7(NR)	4–8 mo	NR	Lumbar spine BMD (CT): ↓−7.5% to −10.8%
Miyamoto [16]	1998	STS	2(1M1F)	8–14 d	Cycle Ergometer	Spine (DEXA): ↓3%, ↓Metacarpal (MD): 1%–3%
Smith [35]	1999	STS/MIR	3M	115 d	NR	↑50% Urinary calcium, ↑50% Collagen crosslink excretion
Vico [36]	2000	MIR	15(14M1W)	1–6 mo	Not Reported	BMD(DEXA):↓1.8% Tibial cortical bone, BMD(DEXA):↓5.4% Tibial cancellous bone
Leblanc [37]	2000	MIR	18(17M1W)	4–14 mo	Bunge Resistance, Treadmill, Cycle Ergometer	Total BMD(DEXA): ↓0.35% per month, Trochanter BMD(DEXA): ↓1.56 per month
Smith [38]	2004	ISS	11(9M2W)	128–195 d	NR	↓25-hydroxycholecalciferol, ↑75% deoxypridinoline, ↑40% n-telopeptide
Smith [39]	2005	STS/MIR	13(12M1F)	4–6 mo	NR	N-telopeptide, pyridinoline, and deoxypyridinoline ↑>55% above preflight levels
	2005	STS/MIR	6M	4–6 mo	NR	Calcium absorption ↓49% in-flight
Lange [40]	2004	ISS	14(13M1W)	4–6 mo	NR	Hip aBMD(DEXA): ↓1.4%–1.5% per month, Spine vBMD(QCT):↓0.9% per month
Smith [31]	2012	ISS	8(6M2W)	48–215 d	CEVIS, TVIS, iRED	Total BMC(DEXA):↓4%, Total BMD(DEXA): ↓3%, Pelvic BMC(DEXA): ↓12%
Smith [31]	2012	ISS	5(3M2F)	48–215 d	CVIS, TVIS, ARED	Total BMC(DEXA):↓1%, Total BMD(DEXA): ↔0%, Pelvic BMC(DEXA): ↓2%

STS = Space transport system, ISS = International space station, CEVIS = Cycle ergometer with isolation system, TVIS = Treadmill with isolation system, iRED = Interim resistive device, VELO = Russian velociped bicycle exercise device, DEXA = Dual x-ray absorptiometry, BMD = Bone mineral density, BMC = Bone mineral content, QCT = Quantitative computer tomography, CT = Computer tomography, d = Days, Yr. = Year, mo = Months, KTF = Exercise device (acronym unknown), M = Men, W = Women, NR = Not reported.

Negative bone adaptations have also been observed more recently after long-duration spaceflight [36]. Grigoriev and colleagues found bone mineral density loss was greatest in the pelvis (−11%), lumbar vertebrae (−6%), and femoral neck (−8%) after 4.5–14.5 month missions [41]. Virtually every astronaut on missions longer than 30 days has lost bone in some region [17]. The extent of bone loss is variable and depends on a variety of factors such as pre-flight bone density, previous exercise experience, diet, and physical activity. On the ISS, average rates of bone loss are 0.9% per month and 1.2%–1.5% per month in the spine and hip, respectively [40]. Perhaps, even more alarming than the loss of bone is the lack of recovery after return to Earth [42]. Of the three Skylab astronauts that lost calcaneus bone, only one showed recovery during the first 90 days post-flight, and all four continued to lose bone for the next five years [15,43]. This lack of recovery or plasticity of bone is important not only for mission success in astronauts participating in multiple flights, but also could enhance fracture, renal stone, and injury risk once back on Earth [2,3,44].

Overall, musculoskeletal alterations during microgravity exposure are moderate in magnitude. Although future missions to Mars, the Moon, or an asteroid may involve periods of exposure to gravitational forces, musculoskeletal health will remain a serious problem for humans both during and after space missions. Exercise and nutrition have been the most widely studied countermeasures to date and remain the primary targets for integration and optimization.

## 3. Exercise in Space

Exercise has been extensively studied and employed to combat disuse-related adaptations that are inherent to the space environment [45]. While early missions of the 1960s did not incorporate in-flight exercise, the next four decades of spaceflight saw a significant emphasis placed on in-flight exercise countermeasures [46]. Exercise hardware evolved from astronauts running in their socks on a slick plate (the Skylab 4 “treadmill”) to advanced equipment such as a fully motorized treadmill with vibration isolation [47] and a strength training device (the Advanced Resistive Exercise Device; ARED) that provides constant forces up to 273 kg with inline flywheels that simulate the inertial forces of weightlifting in normal gravity [48,49]. These advances reflect the complexities of attempting to replicate the physiologic influence of continual exposure to Earth’s gravity which acts even on sedentary (but ambulatory) individuals. Musculoskeletal protection during the early years of the ISS was challenging [9,10,50] with the interim Resistance Exercise Device (iRED) providing only modest loading capability (136 kg) [31]. Improved outcomes have been observed since the deployment of ARED and an updated treadmill [31]. Due to the cost and limited opportunities to conduct exercise countermeasures research during actual spaceflight, bed rest has been used to evaluate the efficacy of a range of exercise countermeasures. 

Bed rest is synonymous with physical inactivity and is employed experimentally as a ground-based analog to more efficiently investigate spaceflight-induced physiological adaptations. This model originated with Soviet scientists who are reported to have raised the foot of the beds of cosmonauts who had recently returned from spaceflight in order to eliminate the cosmonauts’ sensation of slipping off the foot of the bed [51]. Further work by the Soviets found that a 6 degree head down tilt elicited the optimal compromise between comfort and physiologic adaptations; thus, 6 degree head down tilt is the bed rest model most commonly employed in spaceflight research [52]. The cephalid fluid shift induced by this slight head down tilt initiates a systemic diuresis, reducing blood volume ~10%–15% in 1–2 days; this directly mimics what occurs in space as body fluids migrate headward in the absence of Earth’s gravity [51]. Numerous studies have utilized prolonged bed rest to evaluate adaptions and countermeasures. A full detail of these studies is beyond the scope of this review, but many exercise countermeasure studies have included: resistance exercise [53,54,55], aerobic exercise with and without lower body negative pressure [56,57,58], artificial gravity via a centrifuge [59,60], and vibration [61,62]. With the exception of vibration, these countermeasures have been largely successful at the prevention of muscular deconditioning in bed rest, but due to practical issues (e.g., prohibitively large mass, lack of transport vehicles, cost), most have not been similarly implemented in spaceflight. Finally, there is a confounding factor which is somewhat unique to spaceflight that influences exercise training outcomes; spaceflight is associated with a voluntary energy restriction [38] and to date, this important modifying factor has only been included in one bed rest study [63,64].

## 4. Nutrition in Space

The history of nutrition in spaceflight is well-documented [65,66,67,68,69]. With the exception of Skylab 4 where crewmembers consumed 99% of predicted energy requirements, data suggest that crewmembers consume less energy inflight than pre-flight [69]. During Space Shuttle missions, Lane *et al.* [70] determined that on average, energy intake was only 68% of predicted requirements. Similarly, on more recent long-duration ISS missions, energy intake was only ~80% of recommended values [38]. A well-known consequence of negative energy balance during unloading is enhanced muscle protein loss [71], which is largely facilitated by a reduction in muscle protein synthesis [11,72]. Overall, this results in a 5%–10% loss of body mass during spaceflight [38,73].

The U.S. Recommended Dietary Allowance (RDA) for protein is 0.8 g∙kg^−1^∙day^−1^ [74]. However, the RDA for protein in the general population has been critically questioned [73,75] and could underestimate protein needs by as much as 40% [76,77,78]. Studies also demonstrate that protein intakes between 1.2 and 2.0 g∙kg^−1^∙day^−1^ are safe and not associated with renal dysfunction [78,79,80]. In addition, short term (7 day) studies in body builders and athletes suggest that protein intakes up to 2.8 g∙kg^−1^∙day^−1^ failed to correlate with renal impairment as evidenced by creatinine clearance, albumin excretion rate, or calcium excretion rate [80].

Ground-based negative energy balance studies in ambulatory subjects have provided insight into the protective effect of protein on lean mass during periods of reduced energy intake. With traditional caloric restriction diets, a significant portion of total weight loss is lean mass [81]. Demling *et al.* [82] showed that a protein intake of 1.5 g∙kg^−1^∙day^−1^ supported lean mass gain during a 20% energy deficit. In another study, a higher protein diet (1.4 g·kg^−1^·day^−1^) during 12 weeks of caloric restriction was shown to reduce the loss of lean mass (−1.5 kg) compared to a similar isoenergetic diet containing the RDA for protein (−2.8 kg lean mass) [83]. A similar 16 week study evaluated the concomitant effects of different protein intakes with exercise to protect lean tissue during caloric restriction. Together, resistance exercise and a higher protein diet actually increased lean mass during caloric restriction; these results contrasted with those of a control group that consumed a hypocaloric diet containing the RDA for protein and performed the same exercise program but incurred a significant decrease in muscle mass [84]. Importantly, the higher protein group also saw greater increases in strength [84]. Finally, Layman *et al.* evaluated the effects of four dietary groups during caloric restriction: a higher protein diet (1.5 g·kg^−1^·day^−1^), a moderate protein diet consuming the RDA, and each diet combined with resistance exercise [85]. After 16 weeks, all groups experienced significant losses in body mass and fat mass with the greatest losses in the higher protein plus exercise group. Despite having the largest body mass losses, only the higher protein plus exercise group did not lose lean muscle mass [85]. The mechanism underlying these positive results is the critical provision of a threshold amount of leucine at each meal (≥2.5–3 g, equal to the content of ~30 g intact protein) which maximally stimulates muscle protein synthesis, sparing lean tissue during caloric restriction [86]; resistance exercise acts synergistically to enhance this effect [85,86].

In spaceflight, protein intake is recommended to be 12%–15% of total energy consumed at a 60:40 ratio of animal to plant sources [87,88]. On the ISS, protein intake was recently reported to be 102 ± 29 g∙day^−1^ or ~1.4 g∙kg^−1^∙day^−1^ [38] which is consistent with the protein levels now recommended (1.2–1.7 g∙kg^−1^∙day^−1^) for strength and endurance athletes [89,90]. However, the protein recommendations for spaceflight are based on the assumption that total energy needs have been met. As previously discussed, this prerequisite is rarely met during spaceflight [71]. Thus, negative energy balance in the mechanically unloaded environment of space undermines the otherwise adequate daily protein intake, which results in poor muscular outcomes despite the performance of exercise countermeasures. A recent report from spaceflight documented that adequate energy intake with a protein intake of 1.4 g∙kg^−1^∙day^−1^ coupled with exercise countermeasures maintained bone mineral density (or attenuated bone mineral density losses) and increased lean mass during six months on the ISS [31]. Because muscle protein synthetic pathways are blunted during mechanical unloading (e.g., spaceflight, bed rest) [91,92,93], protein intakes between 1.5 and 2.0 g∙kg^−1^∙day^−1^ may be needed during voluntary caloric restriction and exercise to maintain muscle mass during space missions. If energy intake is adequate during spaceflight, the current recommended protein intake (~1.4 g∙kg^−1^∙day^−1^) in combination with advanced exercise countermeasures [49] may be effective [31,73]; however, whether this is optimal remains unknown [94]. This range of protein intake (1.5–2.0 g∙kg^−1^∙day^−1^) is consistent with recent recommendations from the U.S Armed Forces for soldiers to maintain muscle mass and strength during periods of sustained metabolic demand and negative energy balance [95]. Exercise countermeasures (or the absence thereof) also appear to modulate the response to additional dietary protein. In light of recent evidence that demonstrates poor outcomes for muscle with increased protein intake (1.6 g∙kg^−1^∙day^−1^) during unloading in the absence of any exercise countermeasures [55,96], we wish to emphasize the importance of combining exercise countermeasures with increased protein intake for optimal muscular protection during spaceflight. If exercise countermeasures cannot be performed either due to injury or hardware failure, a more modest protein intake of 1.0–1.4 g∙kg^−1^∙day^−1^ may be appropriate to minimize skeletal muscle turnover [55] although this has not been experimentally evaluated in spaceflight. The degree of benefit provided by additional protein in the diet likely depends too on protein quality (*i.e.*, amino acid content). In particular, the essential amino acid leucine has been shown to be a potent stimulant of muscle protein synthesis [97]. Whole foods that contain all of the essential amino acids (leucine, lysine, histidine, phenylalanine, valine, threonine, tryptophan, isoleucine, methionine) are known as complete proteins while those not containing all the essential amino acids are considered incomplete [98]. Also, the rate of intact protein digestion into individual amino acids may influence aminoacidemia and protein accretion [99], which has prompted research into the use of free crystalline amino acids for faster delivery into circulation, especially in combination with exercise [100,101]. 

## 5. Exercise and Amino Acid Supplementation on Earth

Exercise training (resistance and aerobic) is needed as the first line of defense against microgravity-induced musculoskeletal deconditioning as it increases muscle protein synthesis [102] and places mechanical forces on bone to stimulate formation [103]. Skeletal muscle signaling pathways are also very responsive to acute nutritional intake [97]. For example, consumption of free essential amino acids increases the rate of muscle protein synthesis [100,101]. The stimulatory effect of essential amino acids on muscle protein synthesis is enhanced in combination with other potent anabolic stimuli such as exercise [104]. Drummond and Rasmussen reported that resistance exercise alone produced a 40% increase in muscle protein synthesis above fasted levels [97]; intake of essential amino acids and carbohydrate elicited a 100% increase in muscle protein synthesis over the same fasted baseline, while exercise plus essential amino acids and carbohydrate acted synergistically to produce the greatest rise in muscle protein synthesis (145%) [97]. Bird *et al.* [105] showed that resistance exercise training alone increased muscle fiber CSA, but that resistance exercise supplemented with essential amino acids and carbohydrate synergistically improved training adaptations, apparently due to an attenuation of post-exercise muscle protein degradation [105,106]. Together, these reports suggest that resistance exercise and essential amino acid intake enhance muscle protein synthesis and may also inhibit muscle protein breakdown, both of which are needed for optimal muscle protein accretion [72]. The subjects in these studies were not in an energy deficit or mechanically unloaded as observed in space; therefore, it is tenuous to extrapolate these ground-based results to the space environment. The only study to combine exercise and essential amino acid supplementation during mechanical unloading with a mild, 8% energy deficit found that exercise plus essential amino acid supplementation was more effective than essential amino acids alone to protect muscle during bed rest [64]; an exercise-only group was not studied. 

## 6. Protein and Amino Acids for Musculoskeletal Health during Spaceflight: A Paradox?

Evidence suggests that free amino acid ingestion (with or without carbohydrate) and resistance exercise provide a potent, synergistic anabolic stimulus for skeletal muscle [101,107,108,109,110,111,112]. However, there is concern that increasing amino acid intake during spaceflight may increase bone resorption [113,114]. According to the acid-ash hypothesis, diets high in acid-forming substances (anions: amino acids, phosphorus, chlorine) and low in base-forming substances (cations: sodium, potassium, calcium, magnesium) can lead to lower bone mineral density and greater fracture risk [115,116]. Briefly, bone loss occurs because the body requires base and base precursors to buffer acid load. Approximately 80% of total body carbonate is located in the hydrogen shell surrounding bone [117]. Without adequate base or base precursors, carbonate is mobilized from bone to buffer the acid load in the blood [118]. Thus, fruits and vegetables are considered protective to the skeletal system because of their potassium content, while proteins and grains are considered detrimental because of their sulphate and phosphate production [118]. 

There is both scientific support [119,120,121,122] and opposition [118,123,124] to the acid-ash hypothesis as it pertains to the Earth-based general population. It is also not clear how exercise, a stimulus that also alters acid-base balance [125] and is beneficial to bone [103], moderates this relationship. For example, individuals who perform resistance exercise have greater bone mineral densities than those who do not participate in resistance exercise [126] and body builders (a group that is known to consume large amounts of protein and amino acids) have greater bone mineral densities than other athletes that typically do not consume large amounts of protein (e.g., endurance athletes) [127]. 

It can be argued that the unique environment of space causes different metabolic and adaptive responses compared to what is observed on Earth and thus the relationship between protein intake and bone resorption changes. Smith and Zwart have described the potential for a low grade metabolic acidosis during unloading [67] and suggest that the ratio of animal protein to potassium intake predicts the rate of bone resorption during bed rest [67,113]. Similarly, in a 28 day bed rest study, Zwart *et al.* [114] showed that supplementation of essential amino acids (~49.5 g∙day^−1^) and carbohydrate (~90 g∙day^−1^) during 28 days of bed rest had a negative effect on urinary markers of bone breakdown (n-telopeptide and deoxypyridinoline) and calcium. Interestingly, in the same subjects, Paddon-Jones *et al.* [128] reported that supplementation protected participants against decreases in lean mass and muscle protein synthesis. Strength loss was also greater in controls compared to the supplemented group [128]. Essential amino acid supplementation also protects muscle function during bed rest in the elderly [129]. With these apparently opposing adaptations to essential amino acids supplementation in skeletal muscle and bone during unloading, there appears to be a paradox as it pertains to the protection of musculoskeletal health. It is important to note that none of these studies included resistance exercise or reduced energy intake. Currently, only one study provides insight into this multilevel interaction (unloading × essential amino acid supplementation × exercise × reduced energy intake) [63,64]. This 28-day bed rest study reduced energy intake by 8% and assigned participants to one of three groups: (1) essential amino acid supplementation only; (2) resistance training with essential amino acids provided five minutes before training; or (3) resistance training with essential amino acids provided three hours after training. Mid-thigh muscle CSA declined 11% with essential amino acids only, but was maintained in the two groups that performed resistance exercise coupled with essential amino acid supplementation. The greatest loss of lower body muscle strength was also observed with amino acid intake only (−22%), while both resistance exercise plus amino acid supplementation groups showed attenuated strength loss (~7%) [63]. To our knowledge, markers of bone resorption have not been published from the groups in this study, therefore, it is not known if resistance exercise can attenuate the negative bone outcomes that are associated with a low grade metabolic acidosis during unloading. A recent study from the ISS that included exercise countermeasures (Table 2) suggests that higher protein intake was associated with loss of pelvic bone mineral content, but significant relationships were not observed at other bone sites [31]. It should also be noted that the majority of pelvic bone mineral content loss occurred with low intensity iRED resistance exercise countermeasures (−12%) compared to higher intensity ARED resistance exercise countermeasures (−2%), suggesting that exercise intensity may also mediate the effects of protein intake on bone during spaceflight. 

## 7. Perspective and Recommendations for Future Research

Optimizing dietary intake and resistance exercise to preserve both muscle and bone during prolonged spaceflight is a difficult task. Even if energy balance is achieved, the current diet may not optimize the effectiveness of exercise countermeasures for the protection of muscle mass and strength. In the opinion of these authors, the diet is tailored to maintain bone parameters at the expense of skeletal muscle mass (*i.e.*, low acid with Vitamin D and calcium supplementation). We are not attacking this philosophy, particularly given that skeletal muscle is quite plastic, while bone is not. We are simply spotlighting it and wish to emphasize that it will be very difficult, if not impossible, to maintain pre-flight muscle mass levels with a dietary paradigm that emphasizes bone over muscle. Below, we discuss two dietary strategies to enhance muscle parameters without negatively affecting bone. The overall focus of these strategies is to increase total protein intake into the recommended range (1.5–2.0 g∙kg^−1^∙day^−1^) by supplementing free essential amino acids in close temporal proximity to exercise countermeasures. 

### 7.1. Non-Sulfur Containing Essential Amino Acids

The essential amino acids are indispensable to the nutritional stimulation of muscle protein synthesis [100]. However, as previously discussed, increased acidity from amino acids in the diet may lead to increased bone resorption, especially during unloading [114]. Net endogenous acid production from sulfur-containing amino acids (methionine, cysteine, homocysteine, and taurine) are the greatest contributor to decreased blood pH as they can produce sulfuric acid from incomplete oxidation [130]. Of the essential amino acids, only methionine is sulfur-containing [130]. Methionine is also the initiating amino acid in the synthesis of eukaryotic proteins, including skeletal muscle [130]. Therefore, it is critical for translation initiation, but because most residues are subsequently removed, it is not important to protein structure [130]. As such, it may be possible to increase total protein intake to >1.5 g∙kg^−1^∙day^−1^ via non-sulfur-containing essential amino acid supplements in combination with resistance exercise and not see detrimental effects on acid-base balance, particularly if potassium is also supplemented during unloading. Future bed rest or limb suspension research in this area is warranted to elucidate a dietary strategy that is beneficial for both muscle and bone during spaceflight.

### 7.2. Leucine

Because up-mass and volume are always severely limited in spaceflight and will be so particularly for long-duration exploration missions, the mass and volume of a supplement is a key consideration in its potential implementation; for example, a 45 g·day^−1^ essential amino acid supplement (3 × 15 g·day^−1^) equates to 8.1 kg for a single astronaut over a 6-month mission. Ideally, a supplement should be low-volume while still retaining the potent anabolic effects of a full complement of essential amino acids. Over the past 15 years, leucine has emerged as just such an essential amino acid for its singular regulation of muscle protein synthesis [97]. Leucine is a branched-chain amino acid that stimulates muscle protein synthesis *in vitro* [131,132], in rats [133], and in humans [111,134] while the other branched-chain amino acids, valine and isoleucine, are, alone, ineffective and inhibitory, respectively [131]. Together, all three branched-chain amino acids stimulate muscle protein synthesis, but without leucine, the others are ineffective [135]. Leucine exerts this protein synthetic effect by stimulation of mRNA translation initiation [131,136]. Specifically, in rat muscle preparations, leucine-stimulated translation initiation (and protein synthesis) is facilitated by increased binding of the elongation initiation factor 4E (eIF4E), elongation initiation factor 4G (eIF4G) assembly, possibly secondary to increased eIF4G phosphorylation; these effects were seen independent of activation of the mammalian target of rapamycin (mTOR) pathway as phosphorylation of eukaryotic initiation factor (eiF)4E binding protein-1 (4E-BP1) and 70-kDa ribosomal protein S6 kinase 1 (S6K1) was not increased with supraphysiologic levels of leucine [137]. A PI3-kinase inhibitor in this study also clearly demonstrated leucine’s ability to stimulate muscle protein synthesis in the absence of insulin’s effects. Nevertheless, insulin still has potent synergistic effects with leucine on mTOR activation, subsequent translation initiation via phosphorylation of S6K1 and 4E-BP1 and assembly of eIF4G and eIF4E, and ultimately, muscle protein synthesis [133,137,138,139]. 

Leucine has also been shown to stimulate muscle protein synthesis in humans via the mTOR pathway and its downstream targets, S6K1 and 4E-BP1 [140]. Young adults given a 10 g essential amino acid drink with either 1.8 or 3.5 g leucine similarly increased muscle protein synthesis and net balance over a 3 h period. Although synthetic responses were similar, a slightly greater decrease in muscle protein breakdown was seen in the high-leucine group [140]. Further, extra leucine was associated with a prolonged increase in insulin as well as enhanced anabolic signaling, particularly of 4E-BP1 [140]. In light of these findings, leucine has been investigated as a potential intervention to prevent the muscle loss associated with aging and unloading. Verhoeven *et al.* supplemented healthy older men with 2.5 g leucine with each of their three daily meals (7.5 g·day^−1^). After 3 months of supplementation, no changes in lean mass, strength, insulin sensitivity, or lipid profile were observed [141]. They found similar non-effects in a group of older Type II diabetic men who followed the same supplementation regimen for 6 months [142]. However, the subjects in these studies already consumed a moderate protein diet (~1.0 g∙kg^−1^∙day^−1^), were ambulatory, and were not in an energy deficit, which may explain their lack of response to supplemental leucine. Trappe *et al.* also evaluated leucine as a countermeasure during 60 d bed rest in young women, but reported losses in lean mass and strength similar to or worse than controls [55]. It is plausible that leucine may only influence changes at the whole body level in individuals who are in a compromised state (e.g., inactive or consuming a low protein/energy diet). Evidence for this may be found in results from Casperson *et al.* who reported that 14 d supplementation with 4 g leucine meal^−1^ (12 g·day^−1^) in healthy older adults consuming the RDA for protein resulted in increased basal and post-prandial MPS as well as greater mTOR, S6K1, and 4E-BP1 expression [143]. Leucine appears well-suited both to increase total protein intake to 1.5–2.0 g∙kg^−1^∙day^−1^ and to act synergistically with exercise countermeasures to create an optimal anabolic environment during long-duration spaceflight. In contrast to previously discussed supplements containing all the essential amino acids, a leucine supplement could be 10–15 g∙day^−1^, reducing supplement mass or volume by 67% or more. However, further work is needed to evaluate the efficacy of leucine supplementation during unloading and particularly its interaction with negative energy balance and exercise. 

In summary, prolonged exposure to microgravity significantly impairs musculoskeletal health. Exercise is the primary countermeasure against deconditioning; however, spaceflight appears to be associated with a restriction in energy intake which may undermine exercise effectiveness. With negative energy balance and exercise countermeasures, protein intake ≤1.4 g∙kg^−1^∙day^−1^ is likely insufficient to maintain skeletal muscle mass. Adequate total energy intake is the first dietary priority for long-duration spaceflight; however, with negative energy balance and exercise, supplementation of additional protein to achieve intakes of 1.5–2.0 g∙kg^−1^∙day^−1^ (particularly from non-sulfur containing essential amino acids or leucine) may offer secondary protection. Consistent with these recommendations, future unloading studies should evaluate the concomitant effects of non-sulfur containing essential amino acid and/or leucine supplementation with exercise in the context of energy restriction on both skeletal muscle and bone outcomes. 
